# The Combination of the Tumor Markers Suggests the Histological Diagnosis of Lung Cancer

**DOI:** 10.1155/2017/2013989

**Published:** 2017-05-18

**Authors:** Linjie Liu, Jinlong Teng, Lijun Zhang, Peishan Cong, Yuan Yao, Guirong Sun, Zhijun Liu, Teng Yu, Mingjun Liu

**Affiliations:** ^1^Department of Clinical Laboratory, The Affiliated Hospital of Qingdao University, 16 Jiangsu Road, Qingdao 266003, China; ^2^Department of Critical Care Medicine, The Affiliated Hospital of Qingdao University, 16 Jiangsu Road, Qingdao 266003, China; ^3^Department of Microbiology, Weifang Medical University, Weifang, Shandong 261053, China

## Abstract

Tumor markers are beneficial for the diagnosis and therapy monitoring of lung cancer. However, the value of tumor markers in lung cancer histological diagnosis is unknown. In this study, we analyzed the serum levels of six tumor markers (CEA, CYFRA21-1, SCC, NSE, ProGRP, and CA125) in 2097 suspected patients with lung cancer and determined whether the combination of the tumor markers was useful for histological diagnosis of lung cancer. We found that CYFRA21-1 was the most sensitive marker in NSCLC. ProGRP showed a better clinical performance than that of NSE in discriminating between SCLC and NSCLC. The serum level of CYFRA21-1 or SCC was significantly higher in squamous carcinoma (*p* < 0.05), and the levels of ProGRP and NSE were significantly higher in SCLC (*p* < 0.05). According to the criteria established, SCLC and NSCLC were discriminated with sensitivity of 87.12 and 62.63% and specificity of 64.61 and 99.5%, respectively. The sensitivity and specificity in the differentiation of adenocarcinoma and squamous carcinoma were 68.1 and 81.63% and 70.73 and 65.93%, with NPV of 46.03 and 68.97% and PPV of 85.82 and 79.47%, respectively. Our results suggested the combination of six tumor markers could discriminate the histological types of lung cancer.

## 1. Introduction

Lung cancer is the leading cause of cancer-related death globally. It has been the most common incident cancer and identified as leading causes of cancer death in China [1]. Conventionally, lung cancer is divided into two major subtypes: small cell lung cancer (SCLC) and non-small-cell lung cancer (NSCLC). NSCLC is classified into three histological types: adenocarcinoma, squamous cell carcinoma, and large cell carcinoma. SCLC accounts for 15–20% of all lung cancer patients, which is far less than NSCLC patients. SCLC is the worst neoplasm of four histological types. In contrast to NSCLC, SCLC is characterized by its multilocular growth pattern and propensity for early metastases in lymph nodes or distant organs. It has a poorer prognosis than NSCLC. SCLC also differs from NSCLC in treatment by the presence of neuroendocrine differentiation. SCLC shows great sensitivity to chemotherapy and radiotherapy, whereas NSCLC responds well to the traditional surgery way [2–4]. Accordingly, the histological diagnosis of lung cancer is essential for the therapeutic and prognostic implication.

The biopsy is used widely for the histological diagnosis of lung cancer. However, a biopsy is not always convenient, especially in patients with a bad health situation, which is hard for them to bear it. Besides, limited tissue samples and different tissue areas may cause the wrong diagnosis. Therefore, a no-invasion way for the histological diagnosis is required. The circulating tumor markers may be a promising means. A panel of tumor markers has been investigated for their value in lung cancer [5]. Some markers are reliable in the diagnosis and therapy monitoring of lung cancer. For example, high circulating progesterone-releasing peptide (ProGRP) levels have been accepted widely as an indication for SCLC patients. The sensitivity and specificity of serum ProGRP as a tumor marker for SCLC are 60–70% and 96%, respectively [6]. In addition, neuron-specific enolase (NSE) is also a reliable tumor marker for SCLC patients [7]. ProGRP and NSE are not only valuable in the diagnosis of SCLC but also useful in therapy monitoring and for detection of prognosis [8–10]. Carcinoembryonic antigen (CEA), carbohydrate antigen 125 (CA125), squamous cell carcinoma antigen (SCC), and cytokeratin-19 fragments (CYFRA21-1) have been extensively studied in NSCLC [11–16]. CYFRA21-1 is the most sensitive tumor marker in NSCLC [15–20]. And other markers are significant for providing additive information on the histology of lung cancer, such as CEA and CA125 in adenocarcinoma, SCC in squamous tumor, and CA125 in large cell lung cancer [11, 15, 16, 21–24]. The CYFRA21-1 is also reported to be related to squamous cell carcinoma [17, 18]. Above all, these tumor markers may be associated with the lung cancer histological differentiation. However, the utility of tumor markers in lung cancer histological diagnosis is rarely reported.

In this study, we analyzed the serum levels of six tumor markers in suspected patients with lung cancer, evaluated the relationship between tumor markers and lung cancer histological types, and determined whether the combination of the tumor markers was useful for histological diagnosis of lung cancer.

## 2. Material and Methods

### 2.1. Patients

We analyzed the serum levels of six tumor markers in 2097 suspected patients with lung cancer. The suspected patients with lung cancer were evaluated according to the NCCN Clinical Practice Guidelines in Lung Cancer Screening Version 1.2013 [25, 26]. They were finally diagnosed with nonmalignant diseases (126 females and 137 males, age: 56 ± 11.1 years) and with lung cancer (759 females and 1075 males, age 60 ± 9.4 years). The subjects were 2097 suspected patients with lung cancer (1834 cases with lung cancer and 263 cases with benign disease). These patients were from the Affiliated Hospital of Qingdao University between May 2014 and January 2016. The patients with lung cancer included 399 cases with squamous cell carcinoma, 1311 cases with adenocarcinoma, 25 cases with large cell carcinoma, and 99 cases with SCLC. These patients were diagnosed by the tissue biopsy and/or immunohistochemistry according to the 2004 World Health Organization (WHO) classification [27].

### 2.2. Methods

The serum levels of tumor markers were analyzed after diagnosis and prior to treatment. Blood was obtained about 5 mL by venous puncture and centrifuged at 3000 rpm for 15 minutes; the serum was taken and stored in −80°C until assayed (less than 1 month). For all the serum specimens, the serum levels of CEA, NSE, CA125, and CYFRA21-1 were measured by Cobas E601 (Roche, Germany), and the serum level of SCC was determined by Maglumi 4000 (Shenzhen New industries Biomedical Engineering Company, China), and the serum level of ProGRP was analyzed by ARCHITECT i2000 (Abbott, USA).

### 2.3. Cutoff Values

In this study, we took the clinical standard as the cutoff levels of tumor markers. The cutoff levels of CEA and CYFRA21-1 were 5 ng/mL and 3.3 ng/mL, and the cutoff value of SCC, CA125, and ProGRP was 2 ng/mL, 35U/mL, and 50 pg/mL, respectively. As the upper limit of normality, these cutoff values have been applied in clinical for years. We used 25 ng/mL as the NSE cutoff point instead of clinical standard 17 ng/mL.

### 2.4. Statistical Analyses

For statistical analysis, all data were presented as medians, means, and the interquartile range. Nonparametric tests were used for comparisons between numeric variables. The Kruskal-Wallis test, Mann–Whitney *U*, and Student's *t*-tests were used for quantitative results to determine the difference in serum markers between categorical factors or histological subgroup. Statistical calculations were performed using SPSS; a *P* value of less than 0.05 was considered statistically significant. The diagnostic accuracy of serum markers was assessed by plotting receiver operating characteristic (ROC) curves and estimating these markers abilities in discriminating the histology of lung cancer. Sensitivity and specificity as diagnostic test validity were used in the criteria for histological diagnosis. Positive predictive value (PPV) and negative predictive value (NPV) were also obtained.

## 3. Results

### 3.1. The Serum Levels of the Tumor Markers in Patients with Lung Diseases

The serum levels of six tumor markers in 2097 suspected patients with lung cancer (1834 cases with lung cancer and 263 cases with lung benign disease) were shown (Table 1). Significantly higher serum levels were found in patients with NSCLC and SCLC, compared to those with benign disease (*p* < 0.05). The serum levels were moderately elevated in lung benign disease (1.52–13.68%). ProGRP was the most sensitive marker in SCLC (81.82%). Although CYFRA21-1 was the most sensitive marker in NSCLC (32.8%), the percentage of patients with high CYFRA21-1 levels in SCLC was higher than that in NSCLC.

### 3.2. The Serum Levels of the Tumor Markers Were Elevated Differently according to Lung Cancer Histological Types

The relationship between tumor markers and lung cancer histological type was shown (Tables 1 and 2). The serum levels of ProGRP and NSE in SCLC patients were significantly higher compared to NSCLC (*p* < 0.05). The serum levels of CEA, SCC, and CYFRA21-1 were higher in NSCLC than that in SCLC (*p* < 0.05). The serum levels of CYFRA21-1 were significantly higher in squamous carcinoma than that in adenocarcinoma or large cell cancer (*p* < 0.05). The serum levels of SCC and CYFRA21-1 were higher in squamous carcinoma than that in other types (*p* < 0.05). CEA was a sensitive marker in adenocarcinoma and large cell carcinoma. Higher serum levels of NSE and ProGRP were also found in large cell cancer. The abnormal serum levels of CYFRA21-1, CEA, and CA125 were found in both NSCLC and SCLC patients. However, SCLC patients with abnormal serum levels of CYFRA21-1, CEA, and CA125 and normal levels of ProGRP and NSE were rarely found. SCC serum levels were almost normal in SCLC patients.

### 3.3. These Tumor Markers Were Useful in Discriminating the Histological Types

**Figure 1:**
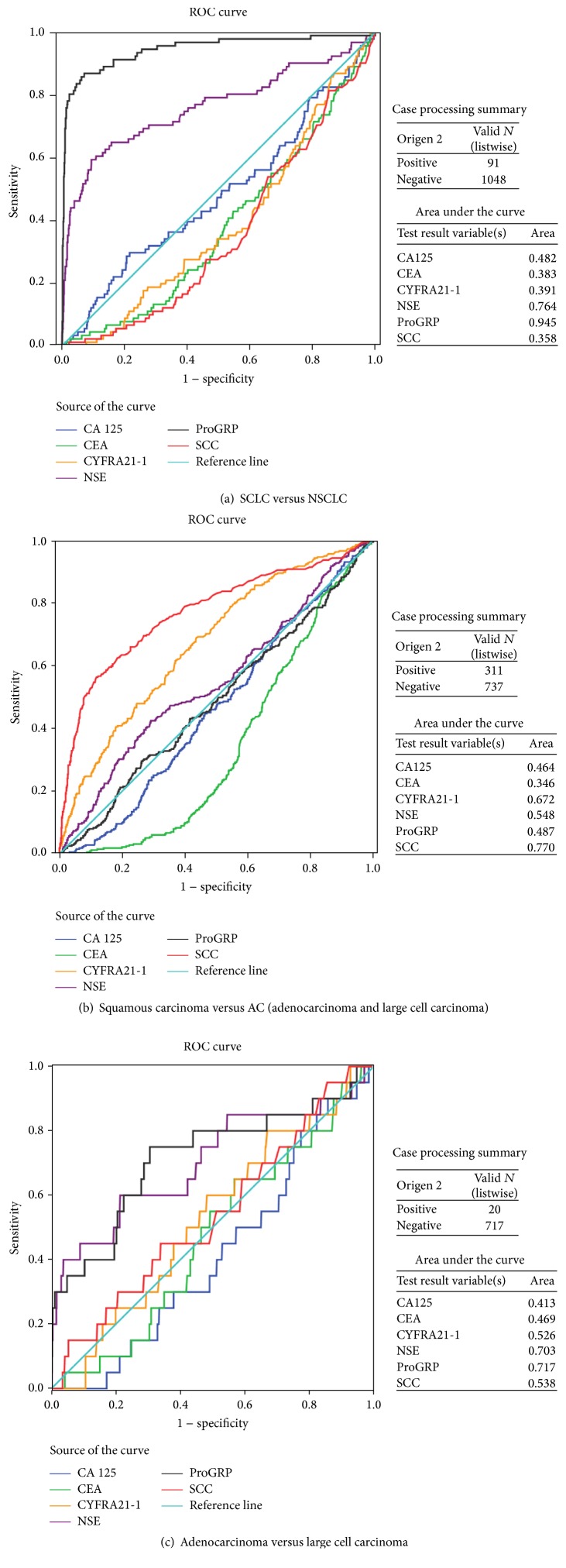
Tumor marker utility in the discrimination of lung cancer subtypes.

The relationship between tumor markers and histological types could be useful for histological diagnosis. ROC curve analysis revealed that ProGRP was better than NSE and the remaining tumor markers in the discrimination between SCLC and NSCLC. The area under the ROC curves (AUC) of ProGRP was significantly greater than that of the other markers (Figure 1).  Table 2 showed the probability of SCLC: the higher the levels of ProGRP and/or NSE, the greater the probability of SCLC. The combination of NSE and ProGRP increased sensitivity and specificity in diagnosing SCLC. SCC was better than the other tumor markers in the discrimination between squamous carcinoma and adenocarcinoma. The AUC of SCC was greater than that for the remaining markers (Figure 1). These tumor markers were not useful in discriminating adenocarcinoma and large cell carcinoma. We classified large cell carcinoma as adenocarcinoma in the present study (Figure 1).

**Figure 2:**
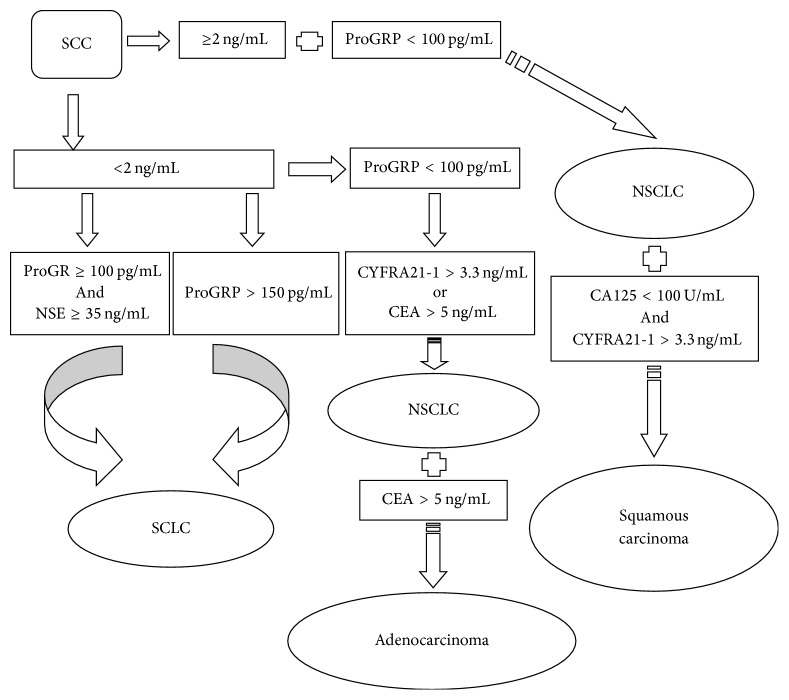
Diagnostic decision tree for lung cancer histological types.

The utility of the combination of these tumor markers in the histological diagnosis of lung cancer was shown in Figure 2 and Table 3. The findings suggested the histological diagnosis of the 62.1% patients (1139/1834), and 93.36% patients (970/1139) were in accord with the results of the biopsy. Sixty-three patients (51 patients with lung benign disease and 12 patients with SCLC) were erroneously classified as NSCLC, including five patients (4 patients with LBD, 1 patient with SCLC) according to criteria 1, thirsty-eight patients (32 patients with LBD, 6 patients with SCLC) according to criteria 2, and twenty patients (15 patients LBD, 5 patients SCLC) according to criteria 3. Besides, six patients (1 patient with LBD, 5 patients with NSCLC) were also erroneously considered as SCLC according to criteria 4 and 5. Furthermore, histological diagnosis criteria of adenocarcinoma and squamous carcinoma were explored. There were 85.81% adenocarcinoma patients (363/423) and 79.47% squamous carcinoma patients (120/151) consistent with the results of the biopsy.

Using these criteria, the value of tumor markers in the histological diagnosis of lung cancer was indicated in Table 4. NSCLC criteria showed a sensitivity of 87.12% and a PPV of 93.55%. SCLC criteria had high specificity (99.5%), PPV (90.47%), and NPV (97.08%). The sensitivity was 68.1 and 81.63%, and specificity was 70.73 and 65.93% in the differentiation of adenocarcinoma and squamous carcinoma, respectively. The sensitivity and specificity in the discrimination of adenocarcinoma and squamous carcinoma were 68.1 and 81.63%, 70.73 and 65.93%, with NPV of 46.03 and 68.97% and PPV of 85.82 and 79.47%, respectively.

## 4. Discussion

Tumor markers are mainly used for monitoring the efficacy of therapy and early detection of recurrence in lung cancer patients. They have been evaluated for their potential in the diagnosis of lung cancer, but none is specific for this malignancy. In this study, we evaluated the relationship between tumor markers and the histological types in lung cancer patients. We modified an algorithm developed by Molina et al. and adapted it for histological diagnosis of NSCLC subtypes without considering the extension of the disease [28]. The sensitivities of all tumor markers in NSCLC were significantly lower than the study of Molina et al. [28]. In the study of Molina et al., the subjects were patients with active lung cancer, whereas the subjects were patients with lung cancer (active and inactive) in our study. The stage and extension of lung cancer could impact the sensitivity of tumor markers. The sample size, race, and regional differences in the patients included in the study could also impact the sensitivities of tumor markers. It is important to present the distribution by stage that could impact the sensitivity of tumor markers. However, we did not collect detailed information on the stage of lung cancer and thus we cannot present the sensitivity by the distribution by stage and analyze the impact of the stage on the sensitivities. We will adopt this important point in our future study on this topic.

We found that CYFRA21-1 was the most sensitive marker in NSCLC, accorded with previous reports [29, 30]. Some studies indicate that pretherapy CYFRA21-1 levels could be used for selection of SCLC patients with an especially poor prognosis [30, 31]. We observed the sensitivity of CYFRA21-1 was lower than that of ProGRP in SCLC. Significantly higher mean serum level and high positive rate of CYFRA21-1 in squamous tumor accorded with previous studies [17, 18]. Abnormal CEA serum levels are both found in NSCLC and SCLC. CEA is associated with adenocarcinoma [21–23, 28, 32]. We found that lung benign disease patients with abnormal CA125 levels mostly had the infectious lung disease. The abnormal SCC serum levels are mainly found in squamous carcinoma.

SCLC is associated with neuroendocrine differentiation [7–9]. NSE is a neuroendocrine marker for SCLC [28, 33, 34]. ProGRP is the most effective circulating marker for SCLC [28, 35]. High ProGRP serum levels are rarely found in lung benign disease and NSCLC [28, 36, 37]. We observed the similar results in this study. ProGRP showed better clinical performance than that for NSE in discriminating between two main lung cancer entities, accorded with previous studies [28, 36]. Our findings suggest that the utility of combination ProGRP and NSE can improve the ProGRP sensitivity in SCLC. Large Cell Neuroendocrine Carcinoma (LCNEC) is no longer classified as a variant of large cell carcinoma. According to the new 2015 WHO classification [38], all lung tumors with neuroendocrine differentiation should be grouped together into one category—neuroendocrine tumors, including those of high grade SCLC and LCNEC, while the diagnosis of large cell carcinoma should be restricted only to small group of resected tumors that lack any clear morphological or immunohistochemical differentiation with reclassification of the remaining former large cell carcinoma subtypes into different categories. Since recent data indicate that chemotherapy regimens used for SCLC may be a better option for LCNEC [39], discrimination of LCNEC from NSCLC has become more relevant. We must adopt the new 2015 WHO classification in our future study on the topic.

Lung cancer histology is important for its implications in the therapy and prognosis [2–4, 34]. Our findings indicate that these markers can differentiate lung cancer histology. The combination of ProGRP and NSE can differentiate SCLC from other types with high sensitivity and specificity; the addition of SCC mainly found in NSCLC is also helpful for the diagnosis of SCLC. It is rarely found in NSCLC patients with an abnormal ProGRP level and SCLC patients with abnormal SCC level. Accordingly, ProGRP and SCC are necessary for the differentiation of SCLC and NSCLC. In the present study, the diagnostic criteria are established for lung histological types without considering the extension of the disease. However, the serum levels of the tumor markers relate to the stage and extension of tumor [28, 30]. We will consider the impact of the stage and extension of the tumor in our future study. The clinical cutoff is a good choice for high sensitivity for locoregional cancer. In the diagnostic criteria, we adopt the upper limits of reference ranges for CYFRA21-1, CEA, and SCC. The cutoff points of 100 or 150 pg/mL for ProGRP and 35 U/mL for NSE provide a high specificity and sensitivity in the diagnosis of SCLC. With these threshold settings, the diagnostic capacity of criteria is stable. We can discriminate SCLC and NSCLC with a high specificity in most patients with lung cancer, and some NSCLC patients are classified as adenocarcinoma or squamous carcinoma correctly. The SCLC criterion for high specificity is more reliable than other criteria. However, these criteria cannot replace the pathological diagnosis. Tumor markers could be useful for some suspected patients with lung cancer in whom the serum markers are the only choice that limited by their health situation, or as one of diagnostic tools in patients who has other clinical evidences indicating the histology.

In conclusion, these tumor markers are related to lung cancer histological types. Using the diagnostic criteria, SCLC and NSCLC are discriminated with a high specificity in most patients with lung cancer. The combination of six tumor markers could explore its value in histological diagnosis in patients with lung cancer.

## Figures and Tables

**Table 1:** Tumor marker serum levels in 2097 patients with lung disease.

Patients	Lung benign diseases	NSCLC	SCLC	Squamous carcinoma	Adenocarcinoma	Large cell carcinoma
	263	1735	99	399	1311	25
CEA > 5 ng/mL	17 (6.46%)	495 (28.53%)	25 (25.25%)	86 (21.55%)	398 (30.05%)	11 (44%)
Mean ± SD	2.22 ± 1.98	18.43 ± 82.08	13.85 ± 65.23	5.04 ± 14.32	22.58 ± 93.63	14.46 ± 40.22
Median	1.79	2.85	2.85	2.93	2.80	4.64
95th percentile	5.37	71.06	30.66	10.98	92.44	154.41

CA125 > 35 U/mL	35 (13.31%)	317 (18.27%)	29 (29.29%)	80 (20.05%)	234 (17.84%)	3 (12%)
Mean ± SD	20.77 ± 35.45	36.46 ± 151.09	39.77 ± 60.28	26.41 ± 36.29	39.79 ± 172.53	22.19 ± 16.21
Median	11.42	13.71	16.80	15.16	13.28	17.52
95th percentile	67.90	117.04	129.30	79.38	131.32	64.52

SCC > 2 ng/mL	4 (1.52%)	233 (13.43%)	5 (5.05%)	147 (36.68%)	83 (6.33%)	3 (12%)
Mean ± SD	0.69 ± 0.46	1.48 ± 4.22	0.82 ± 1.61	3.40 ± 7.98	0.92 ± 1.65	0.92 ± 0.78
Median	0.56	0.66	0.56	1.38	0.61	0.62
95th percentile	1.62	4.02	2.19	13.75	2.24	2.91

CYFRA21-1 > 3.3 ng/mL	31 (11.78%)	569 (32.8%)	40 (40.40%)	248 (62.15%)	399 (30.43%)	12 (48%)
Mean ± SD	2.25 ± 1.34	4.6 ± 10.51	3.38 ± 2.04	7.72 ± 14.32	3.65 ± 8.95	3.63 ± 1.95
Median	1.99	2.68	2.74	3.95	2.39	3.19
95th percentile	4.35	12.15	7.27	22.14	8.37	7.92

NSE > 25 ng/mL	7 (2.66%)	83 (4.78%)	48 (50%)	30 (7.51%)	45 (3.43%)	8 (32%)
Mean ± SD	14.09 ± 8.08	15.51 ± 10.06	45.48 ± 56.63	16.61 ± 8.23	14.77 ± 7.66	36.73 ± 49.69
Median	12.99	13.87	24.71	14.23	13.72	17.47
95th percentile	19.86	24.76	184.20	29.09	22.80	189.96

ProGRP > 50 pg/mL	22 (8.37%)	140 (8.07%)	81 (81.82%)	33 (8.27%)	99 (7.55%)	8 (32%)
Mean ± SD	31.14 ± 13.29	39 ± 160.28	931.2 ± 1364.54	33.15 ± 20.46	35.46 ± 118.08	318.2 ± 1002.76
Median	31.14	29.45	281.56	29.47	29.39	40.37
95th percentile	55.04	55.97	4451.90	56.36	54.57	3817.24

**Table 2:** Probability of SCLC according to NSE and/or ProGRP serum levels.

Criteria	Probability (SCLC/total lung cancer)
ProGRP > 50 pg/mL	33.33% (81/243)
ProGRP > 75 pg/mL	71.56% (73/102)
ProGRP > 100 pg/mL	82.5% (66/80)
ProGRP > 125 pg/mL	84.42% (65/77)
ProGRP > 150 pg/mL	85.71% (60/70)
NSE > 25 ng/mL	34.78% (48/138)
NSE > 30 ng/mL	47.72% (42/88)
NSE > 35 ng/mL	55.07% (38/69)
NSE > 40 ng/mL	58.82% (30/51)
NSE > 45 ng/mL	56.81% (25/44)
ProGRP > 100 pg/mL and NSE > 35 ng/mL	92.11% (35/38)

**Table 3:** The criteria of lung cancer histological diagnosis.

	Criteria	Correct classification/total
NSCLC	SCC ≥ 2 ng/mL and ProGRP < 100 pg/mL [1] SCC < 2 ng/mL and ProGRP < 100 pg/mL and CYFRA21-1 > 3.3 ng/mL [2] SCC < 2 ng/mL and ProGRP < 100 pg/mL and CEA > 5 ng/mL [3]	233/238 (97.89%) 492/530 (92.83%) 403/423 (95.27%)

SCLC	SCC < 2 ng/mL and ProGRP ≥ 100 pg/mL and NSE ≥ 35 ng/mL [4] SCC < 2 ng/mL and ProGRP > 150 pg/mL [5]	32/35 (91.42%) 57/63 (90.48%)

Adenocarcinoma	SCC < 2 ng/mL and ProGRP < 100 pg/mL and CEA > 5 ng/mL [3]	363/423 (85.81%)

Squamous carcinoma	[1] and CA125 < 100 U/mL and CYFRA21-1 ≥ 3.3 ng/mL [6]	120/151 (79.47%)

**Table 4:** The diagnostic value of criteria in discriminating histological types.

	Sensitivity	Specificity	PPV	NPV
NSCLC	87.12% (913/1048)	64.61% (115/178)	93.55% (913/976)	46% (115/250)
SCLC	62.63% (57/91)	99.5% (1129/1135)	90.47% (57/63)	97.08% (1129/1163)
Adenocarcinoma	68.1% (363/533)	70.73% (145/205)	85.82% (363/423)	46.03% (145/315)
Squamous carcinoma	81.63% (120/147)	65.93% (60/91)	79.47% (120/151)	68.97% (60/87)

NSCLC versus Other 1; SCLC versus Other 2; adenocarcinoma versus Other 3; squamous carcinoma versus Other 4. Other 1 = SCLC and benign disease; Other 2 = NSCLC and benign disease; Other 3 = squamous carcinoma, large cell carcinoma, and benign disease; Other 4 = adenocarcinoma, large cell carcinoma, and benign disease.
